# Endoplasmic reticulum-associated SARS-CoV-2 ORF3a elicits heightened cytopathic effects despite robust ER-associated degradation

**DOI:** 10.1128/mbio.03030-23

**Published:** 2023-12-11

**Authors:** Jiantao Zhang, Ruth Cruz-Cosme, Chenyu Zhang, Dongxiao Liu, Qiyi Tang, Richard Y. Zhao

**Affiliations:** 1 Department of Pathology, University of Maryland School of Medicine, Baltimore, Maryland, USA; 2 Department of Microbiology, Howard University College of Medicine, Washington, DC, USA; 3 Department of Microbiology and Immunology, University of Maryland School of Medicine, Baltimore, Maryland, USA; 4 Institute of Human Virology, University of Maryland School of Medicine, Baltimore, Maryland, USA; 5 Institute of Global Health, University of Maryland School of Medicine, Baltimore, Maryland, USA; 6 Research & Development Service, VA Maryland Health Care System, Baltimore, Maryland, USA; Johns Hopkins Bloomberg School of Public Health, Baltimore, Maryland, USA

**Keywords:** SARS-CoV-2, ORF3a mutants, ER and lysosomes, apoptosis, NF-kB, TNFα and IL-6, ER stress, autophagy, reticulophagy, ER-associated degradation, TRIM59, 26S proteasome

## Abstract

Severe acute respiratory syndrome coronavirus 2 (SARS-CoV-2) ORF3a protein plays a vital role in viral pathogenesis and coronavirus disease 2019 (COVID-19). Like the spike protein, ORF3a mutates frequently, and certain variants are associated with the severity of COVID-19. Given the clinical significance and functional implications of ORF3a mutations, we conducted a comprehensive mutagenesis study targeting various known functional elements and revealed two distinctive types of ORF3a proteins based on their subcellular localizations: ORF3a proteins primarily localize on the lysosomal membrane (L-ORF3a) and those present in the endoplasmic reticulum (E-ORF3a). The objective of this study was to contrast the functional and mechanistic distinctions between these two types of ORF3a proteins. We examined six distinct ORF3a mutants and assessed their effects on cellular oxidative stress, nuclear factor kappa B-induced cytokine production, and cell death. Mechanistically, we explored ORF3a-induced ER stress, autophagy, and interactions with relevant cellular proteins. Our findings indicate that ORF3a proteins induce cytopathic effects through a similar mechanism, irrespective of their subcellular location. However, E-ORF3a proteins elicit more pronounced cytopathic effects despite their lower abundance and minimal impact on ER stress and autophagy when compared to L-ORF3a proteins. This discrepancy is attributed to ER-associated degradation since ORF3a proteins bind to a ubiquitin E3 ligase TRIM59. Inhibition of the 26S proteasome partially restores the protein levels of E-ORF3a and cellular ER stress response. This suggests that even a small quantity of ORF3a can lead to significant cytopathic effects due to the delicate nature of ER. Our study underscores the intricate interplay of dynamic cellular signaling within these two subcellular compartments in response to ORF3a.

**IMPORTANCE:**

The severe acute respiratory syndrome coronavirus 2 (SARS-CoV-2) pandemic has tragically claimed millions of lives through coronavirus disease 2019 (COVID-19), and there remains a critical gap in our understanding of the precise molecular mechanisms responsible for the associated fatality. One key viral factor of interest is the SARS-CoV-2 ORF3a protein, which has been identified as a potent inducer of host cellular proinflammatory responses capable of triggering the catastrophic cytokine storm, a primary contributor to COVID-19-related deaths. Moreover, ORF3a, much like the spike protein, exhibits a propensity for frequent mutations, with certain variants linked to the severity of COVID-19. Our previous research unveiled two distinct types of ORF3a mutant proteins, categorized by their subcellular localizations, setting the stage for a comparative investigation into the functional and mechanistic disparities between these two types of ORF3a variants. Given the clinical significance and functional implications of the natural ORF3a mutations, the findings of this study promise to provide invaluable insights into the potential roles undertaken by these mutant ORF3a proteins in the pathogenesis of COVID-19.

## INTRODUCTION

The coronavirus disease 2019 (COVID-19) pandemic caused by severe acute respiratory syndrome coronavirus 2 (SARS-CoV-2) has prompted extensive investigation into the molecular mechanisms behind the pathogenesis of this viral infection. Among the intricate ensemble of viral proteins, ORF3a has emerged as a critical player in viral pathogenesis and the severity of COVID-19. For comprehensive insights into ORF3a’s role in viral pathogenesis and its impact on the severity of COVID-19, refer to the reviews by (1, 2).

The ORF3a protein consists of 275 amino acids (aa) and with a calculated molecular weight of 31 kDa (1). It has been identified to form homodimers or tetramers. Within each monomer, three transmembrane (TM) domains traverse the membrane and cytosol, while distinct functional domains are responsible for diverse functionalities, including ion channel formation, intracellular transport, cytopathic effect, virus release, and virus production (1, 3–5).

ORF3a is unique to SARS-CoV and SARS-CoV-2 among the seven known human coronaviruses (6), which indicates the clinical importance of ORF3a in causing severe human diseases like SARS or COVID-19. Indeed, clinical investigations have unveiled a significant presence of anti-ORF3a antibodies in COVID-19 patients, with sera from these patients exhibiting heightened levels of IgG and IgA reactivity specifically against ORF3a (7, 8). Furthermore, several studies have linked ORF3a to the severity and fatality of COVID-19 (9–13). Supporting clinical observations, *in vitro* studies using SARS-CoV-2 infected cells or cells expressing ORF3a alone have demonstrated that ORF3a triggers a proinflammatory immune response, leading to NLRP3 inflammasome activation—a key contributor to the cytokine storm, responsible for COVID-19-related deaths (9–13). Additionally, we, along with others, have shown that expression of ORF3a in lung and kidney epithelial cells triggers host cellular innate oxidative stress and proinflammatory immune responses (14–16) and induces apoptotic cell death through the production of reactive oxygen species (ROS) mediated by cellular oxidative stress, as well as nuclear factor kappa B (NF-kB)-mediated TNFα (tumor necrosis factor-alpha) and interlukin-6 (IL-6) cytokine production (14, 15, 17), which are two strong and independent predictors of COVID-19-related death (18, 19).

ORF3a is a membrane-associated viroporin. Upon sub-genomic transcription of SARS-CoV-2 structural and accessary proteins, the ORF3a protein is synthesized in the endoplasmic reticulum (ER) (20). After ORF3a protein is produced, it is exported from ER to the Golgi apparatus, where it undergoes posttranslational modification of O-glycosylation before being inserted into the plasma membrane and endomembranes including endosomes and lysosomes (21, 22). Hence, there is a dynamic process for intracellular movement of ORF3a protein post-ORF3a protein production. Functional domains present in ORF3a protein like the YXXΦ motif (aa 160–163) and the diG motif (aa 187–188) at the cytoplasmic region drive ORF3a’s transport from ER to plasma membranes and other endomembranes (5, 23, 24).

While present at ER, ORF3a causes cellular ER stress response (25–27). Once ORF3a arrives at lysosomes, it triggers a host cellular antiviral autophagy response, resulting in the activation of the HOPS complex. This complex serves as a bridge between closely juxtaposed autophagosomal and lysosomal membranes, facilitating the fusion of autophagosomes with lysosomes (25, 28). In order to counteract the host cellular antiviral autophagy response, ORF3a interacts with VPS39, a component of the HOPS complex, thereby preventing the fusion of autophagosomes with lysosomes (29, 30). As a result, it induces host cellular innate oxidative stress and proinflammatory immune responses leading to apoptotic cell death (14). Additionally, there is a crosstalk between ER stress and autophagy, a process that is known as reticulophagy or ER-phagy where ER stress elicits a selective form of autophagy, aka ER-phagy, that transpires ER-associated degradation (ERAD) leading to degradation of ER-residing proteins or ER destruction (31). Early studies showed that induction of reticulophagy by SARS-CoV-2 is essential for viral infection and replication, and ORF3a elicits reticulophagy response and then disrupts ER homeostasis to induce ER stress and inflammatory responses during SARS-CoV-2 infection (25–27).

Similar to the spike protein, ORF3a also undergoes frequent mutations. During the course of the pandemic, numerous natural ORF3a variants have been identified in association with the virus of interest or virus of concern as defined by the World Health Organization (1). This raises the possibility that these ORF3a variants might contribute to alterations in viral virulence and the pathogenesis of COVID-19. Indeed, some ORF3a mutations have been linked to the severity of COVID-19, while others have been associated with fatal outcomes (32–34). For instance, mutations like Q57H, G251V, and S253P have been associated with severe outcomes, including hospitalization and fatality, with the S253P mutation linked to deadly outcomes (32). In a separate study, out of 70,752 screened SARS-CoV-2 ORF3a variants, 17 unique variants were identified. Ten of these variants are located in the TM domains, while the other seven mutations are found in the extracellular N-terminus or the C-terminal cytoplasmic β-sheet domains (35). However, it remains unknown whether these natural ORF3a variants affect the function of ORF3a. An objective of this study was to study the function-structure relationship of ORF3a through mutagenesis. Previously, we carried out a comprehensive mutagenesis investigation by characterizing a panel of 16 functionally relevant ORF3a mutants including single aa and deletion mutations that are in the putative function-relevant motifs and other regions of interest (5). Through that study that was expanded in this study, we examined subcellular localization of those ORF3a mutants in pulmonary and renal epithelial cell lines and uncovered two distinct types of ORF3a mutations based on their subcellular localizations, i.e., the lysosomal membrane-associated ORF3a proteins that we termed as the L-ORF3a protein and the ORF3a proteins that are present in the ER that we coined as the E-ORF3a protein. Notably, some of those E-ORF3a proteins that reside in ER are naturally occurring ORF3a mutants such as the Y233N mutant or the G188 deletion mutant (∆G188) where several natural mutant variants (G188C/D/V) were present, and this glycine residue is critical for intracellular transport of ORF3a (1, 5, 6, 14). However, the functional differences of these two types of ORF3a mutant proteins on host cellular responses and functional outcomes remain unexplored. Given the clinical relevance and functional implications of ORF3a, it is crucial to gain a deeper understanding of the effect of structurally defective or natural ORF3a mutations on their functionalities and their impact on viral pathogenesis and COVID-19. Therefore, the objective of this study was to expand our early mutational analysis and conduct a comparative and functional study between ORF3a in these two subcellular locations. Specifically, we aimed to decipher the differences of these two types of ORF3a proteins in their abilities to induce cellular oxidative stress and proinflammatory immune responses as well as their functional consequences. To gain deeper insights into the underlying molecular mechanisms, we also investigated ORF3a-induced cellular autophagy, ER stress responses, reticulophagy, the dialog between lysosomes and ER, as well as ORF3a interactions with relevant cellular proteins.

## RESULTS

### Mutations in ORF3a proteins result in two distinct groups of proteins characterized by their subcellular distributions: the lysosome-associated ORF3a and the ER-associated ORF3a

**Fig 1:**
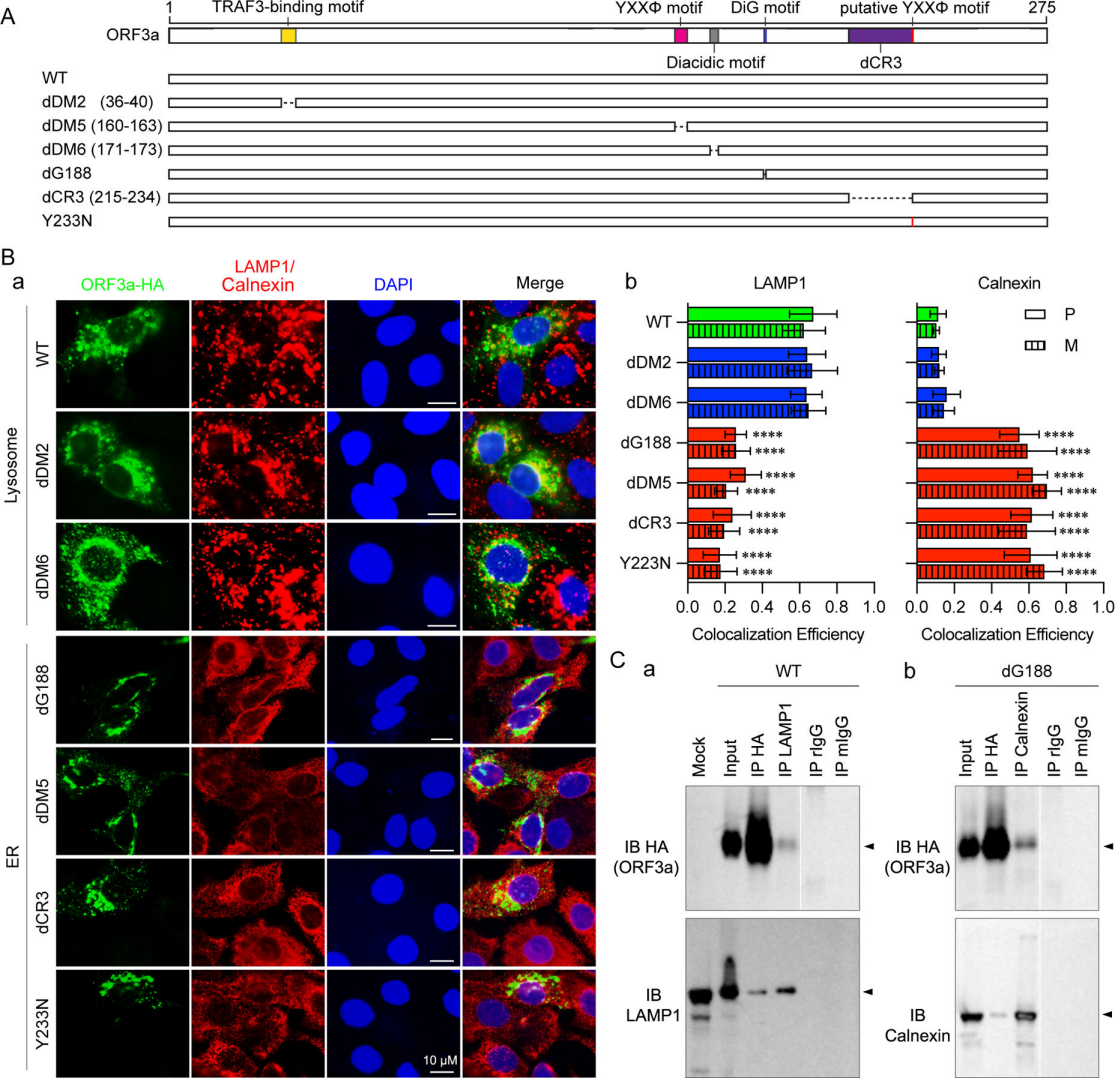
Distinctive subcellular localization and protein interaction patterns of ORF3a proteins. (**A**) Schematic diagram showing the selected ORF3a mutant panel used in this study. For detailed description of these mutants, see reference 5. (**B**) Subcellular localization analysis by IFA (**A**) depicting subcellular localizations of ORF3a proteins in lysosomes or ER. Similar to WT ORF3a, other mutants (dDM2:∆aa36-40 and dDM6:∆aa171-173) exhibit substantial presence within lysosomes, represented by merged images of green-labeled ORF3a and red-labeled lysosomal membrane protein LAMP-1, appearing as characteristic cytoplasmic circular vesicle structures. Blue DAPI staining indicates nuclei. HK2 cells were transfected with a HA-tagged *ORF3a*-carrying plasmid for 24 h. Transfected cells were fixed and permeabilized for IFA using anti-HA antibodies to visualize ORF3a or its mutants in green, and anti-LAMP-1 to show lysosomes or anti-Calnexin to show ER in red. Scale bar: 10 µM. (**B**) Quantitative analysis of ORF3a co-localization with lysosomes or ER using ImageJ2 and JACoP plugin analyses. Pearson’s correlation coefficients (**P**) and Mander’s overlap coefficients (**M**) were calculated. Mean and standard deviation of *P*- and *M* values were obtained from at least 10 random images. Two-way ANOVA was performed, and each mutant was compared with WT to evaluate the level of significance. Statistical significance was considered at the 95% confidence level of *P* < 0.05 (*), *P* < 0.01 (**), *P* < 0.001 (***), and *P* < 0.0001 (****), respectively. (**C**) Protein interaction analysis by reciprocal co-IP analysis (**A**) demonstrating interaction of WT ORF3a with lysosomal membrane-associated LAMP-1. 293T cells were transfected with WT or dG188 mutant ORF3a-carrying plasmid DNA. At 24 hpt, transfected cells were lysed for co-IP analyses. Anti-HA pulldown precipitates the C-terminal HA-tagged ORF3a protein (~32 kDa), confirmed by anti-HA antibody. The presence of LAMP-1 protein (45 kDa) in pulldown products was verified by anti-LAMP-1 antibody. Reciprocal IP experiment using anti-LAMP-1 pulldown. The presence of LAMP-1 protein and ORF3a in pulldown products was confirmed by anti-LAMP-1 and anti-HA antibodies, respectively. Rabbit IgG (rIgG) and mouse IgG (mIgG) are negative controls and used to eliminate possible background signals generated by rabbit and mouse antibodies used for IP. Note that the level of pull-down of LAMP1 or Calnexin depends upon the level of its expression, quality of antibody, and the number of cells used rather than the amount of ORF3a. Consequently, this assay cannot be used as a quantitative measure to correlate with the *M*/*P* values. All information provided is from the same gel. The division between IP LAMP-1 and IP rlgG is intentional to indicate that other irrelevant lanes have been removed. (**B**) Similar reciprocal IP experiments were conducted with anti-Calnexin antibody to assess interaction of the dG188 mutant ORF3a protein with ER-specific Calnexin protein (67 kDa) (38). Arrows indicate the protein of interest.

Built upon our prior mutagenesis studies carried out in lung epithelial cells (5), our goal here was to further investigate the intracellular localization of selected mutant ORF3a proteins (Fig. 1A) across different cell lines. To accomplish this, we employed an immunofluorescence assay (IFA) to detect the ORF3a protein and its association with specific organelles, including lysosomes and the ER. Specifically, an ORF3a-bearing plasmid producing a HA-tagged ORF3a protein was introduced into renal proximal tubular epithelial HK2 cells. At 24 h post-transfection (hpt), cells were fixed and subjected to IFA using an anti-HA antibody. Organelle-specific antibodies targeting lysosome-associated membrane protein 1 (LAMP-1) and Calnexin were used to identify ORF3a’s presence in lysosomes, and ER, respectively. Furthermore, we quantified the extent of ORF3a co-localization with each organelle using ImageJ and JACoP analyses, presenting the results through Pearson’s correlation coefficients (*P* value) or Mander’s overlap coefficients (*M* value) (36, 37). Figure 1B–a displays representative images, with quantification shown in Fig. 1B–b.

In Fig. 1B–a, HA-tagged ORF3a proteins are depicted in green, while LAMP-1 or Calnexin is shown in red, and cell nuclei are stained blue with DAPI. Yellow areas indicate co-localization of ORF3a with lysosomes or ER when these images are merged; otherwise, ORF3a remains green. As we previously reported (5), mutant ORF3a proteins can be distinctly categorized into two groups based on their primary subcellular localization compared to wild-type (WT) ORF3a. In this study, besides the WT control, the dDM2:∆aa36-40 and dDM6:∆aa171-173 mutants were selected to represent the L-ORF3a protein in subsequent studies. This choice was based on the fact that the aa36–40 region serves as a TNF receptor-associated factor 3 (TRAF3)-binding motif, which is known to be associated with ORF3a-induced activation of NF-κB and NLRP3 inflammasomes (10, 39). Furthermore, the dDM6:∆aa171-173 mutant deletes a possible binding site of ORF3a to VPS39, which is a critical part of the homotypic fusion and vacuole protein sorting (HOPS) complex for the induction of autophagy (29, 30, 40). For the representation of E-ORF3a proteins, the dG188 and dDM5:∆aa160-163 mutants were chosen. These mutants were selected because they harbor mutations in the YXXΦ motif (aa160-163) and the diG motif (aa187-188), respectively, rendering them defective in the transport of ORF3a from the ER to lysosomes (5, 23, 24). Additionally, the dCR3:∆aa215-234 mutant was chosen to represent E-ORF3a. This choice was made not only because this mutant primarily resides in the ER and lacks a known functional motif but also because this region encompasses naturally occurring mutations such as Y233N and T223I, with the latter being identified in the Omicron variants (1). Bar graphs in Fig. 1B–b illustrate the relative abundance of ORF3a in the lysosomes or ER, represented by P and M values. These values convey the average percentage of ORF3a correlated or overlapped with the lysosomes or ER, respectively. Similar to our previous findings in lung epithelial A549 and Calu-3 cells (5), L-ORF3a proteins co-localize predominantly with LAMP-1 with *P* values ranging from approximately 0.64 to 0.67 (Fig. 1B–b, left). Conversely, E-proteins primarily associate with Calnexin with *P* values ranging from 0.55 to 0.62 (Fig. 1B–b, right). A similar trend is observed in the *M* values.

To determine whether ORF3a proteins within lysosomes or the ER are membrane-bound due to interactions with lysosomal or ER membranes, we conducted reciprocal co-immunoprecipitation (co-IP) experiments in kidney epithelial 293T cells. These experiments aimed to confirm potential and specific interactions between L-ORF3a proteins, represented by WT ORF3a, with LAMP-1, and E-ORF3a proteins, represented by the dG188 mutant, with Calnexin, both of which are lysosomal and ER membrane-associated proteins. Initially, we pulled down HA-tagged ORF3a proteins using anti-HA antibodies and subjected them to SDS-PAGE analysis. Figure 1C–a shows a prominent protein band detected by the anti-HA antibody at the expected size of approximately 31 kDa. In contrast, no signal was observed in mock, rabbit IgG (rIgG), and mouse IgG (mIgG) pulldowns, which are used as negative controls, indicating successful and specific HA-ORF3a pulldown. Subsequent immune-blotting (IB) using the anti-LAMP1 antibody revealed a distinct protein band around 45 kDa, confirming the interaction with LAMP-1. Similarly, when anti-LAMP-1 antibody was used for the pulldown (Fig. 1C–a), the same 45 kDa protein band was identified in the precipitated proteins, as confirmed by the anti-LAMP-1 antibody. This supports the notion that WT ORF3a not only localizes within lysosomes but also interacts with lysosomal membranes through LAMP-1. Comparable co-IP experiments conducted on the dG188 mutant also validated the interaction of dG188 with Calnexin, a biomarker of the ER membrane (Fig. 1C–b). Note that the level of pull-down of LAMP1 or Calnexin depends upon the level of its expression, quality of antibody, and the number of cells used rather than the amount of ORF3a. Consequently, the IP result cannot be used as a quantitative measure to correlate with the *M*/*P* values shown in Fig. 1A.

In summary, the data presented here support the notion that mutant ORF3a proteins segregate into two distinct groups based on their primary subcellular localization: L-ORF3a (lysosome-associated) and E-ORF3a (ER-associated) proteins.

### ORF3a proteins in the ER induce enhanced apoptotic cell death compared to those localized on lysosomes

**Fig 2:**
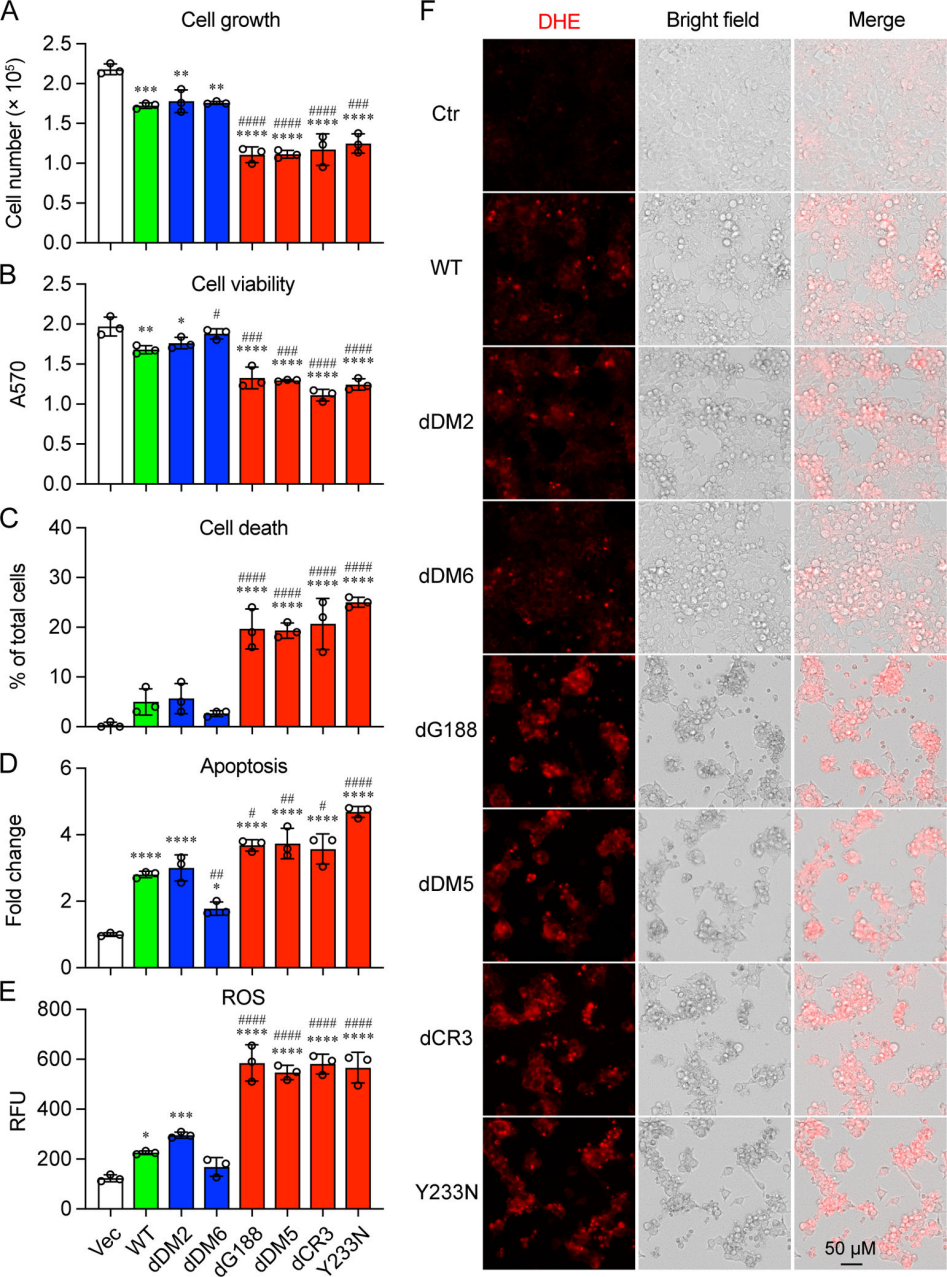
Enhanced apoptotic cell death induced by E-ORF3a proteins compared to L-ORF3a proteins. 293T cells were transfected with a pCAG plasmid expressing either L-ORF3a or E-ORF3 protein variants. After 48 hpt, transfected cells were collected and analyzed on cellular (**A**) growth, (**B**) viability, and (**C**) cell death, assessed through cell counting, MTT assay, and trypan blue staining, respectively. ORF3a-triggered apoptosis (**D**) was determined using a RealTime-Glo annexin V apoptosis and necrosis assay (Promega; Cat# JA1011). Cellular oxidative stress induced by ORF3a (**E**) was assessed by quantifying ROS production through a ROS detection cell-based assay kit (Cayman Chemical; Cat# 601290) with images shown in (**F**). Scale bar, 50 µM. The data, represented as mean ± SE, derive from three independent experiments. Statistical analysis employed pairwise *t*-tests, with significance denoted as follows: * or #, *P* < 0.05; ** or ##, *P* < 0.01; *** or ###, *P* < 0.001; and **** or ####, *P* < 0.0001, respectively. Comparisons were made between Vec and each ORF3a protein, indicated by *, or between WT ORF3a and other mutant ORF3a proteins, indicated by #, respectively.

Building upon our previous findings that ORF3a induces apoptotic cell death partly through the induction of cellular innate oxidative stress response (14), we further investigated the potential differences in the ability of these two types of ORF3a proteins to induce oxidative stress and cell death. To conduct the test, we transfected 293T cells with a pCAG plasmid expressing either L-ORF3a or E-ORF3a protein variants. At 48 hpt, we collected transfected cells and assessed cellular growth (Fig. 2A), cell viability (Fig. 2B), and cell death (Fig. 2C) through cell counting, MTT assay, and trypan blue staining, respectively. ORF3a-induced apoptosis (Fig. 2D) was determined using a RealTime-Glo annexin V apoptosis and necrosis assay. Additionally, we evaluated cellular oxidative stress induced by ORF3a (Fig. 2E and F) by quantifying ROS production via a ROS detection cell-based assay kit. As depicted in Fig. 2, the expression of L-ORF3a proteins (shown in blue bars) exhibited similar levels of reduced cell growth, cell viability, and apoptotic cell death when compared to WT ORF3a (in green bars). In contrast, the expression of E-ORF3a proteins (in red bars) resulted in more potent cytopathic effect as measured by cellular growth, viability, and apoptosis, with statistically significant differences (*P* < 0.001 to *P* < 0.0001). Furthermore, the expression of E-ORF3a proteins also induced much stronger cellular oxidative stress responses than L-proteins, as evidenced by DHE staining (Fig. 2F) and quantified in Fig. 2E.

These findings suggest that ORF3a proteins in the ER are more potent inducers of apoptotic cell death compared to ORF3a proteins localized on lysosomes. It is worth noting that the dDM6:∆aa171-173 mutant’s ability to induce cell death was further reduced, among the L-ORF3a proteins. The significance of this reduction is evaluated in the following section.

### ORF3a proteins residing in the ER elicit significantly higher NF-kB activation and elevated cytokine production than those localized in lysosomes

**Fig 3:**
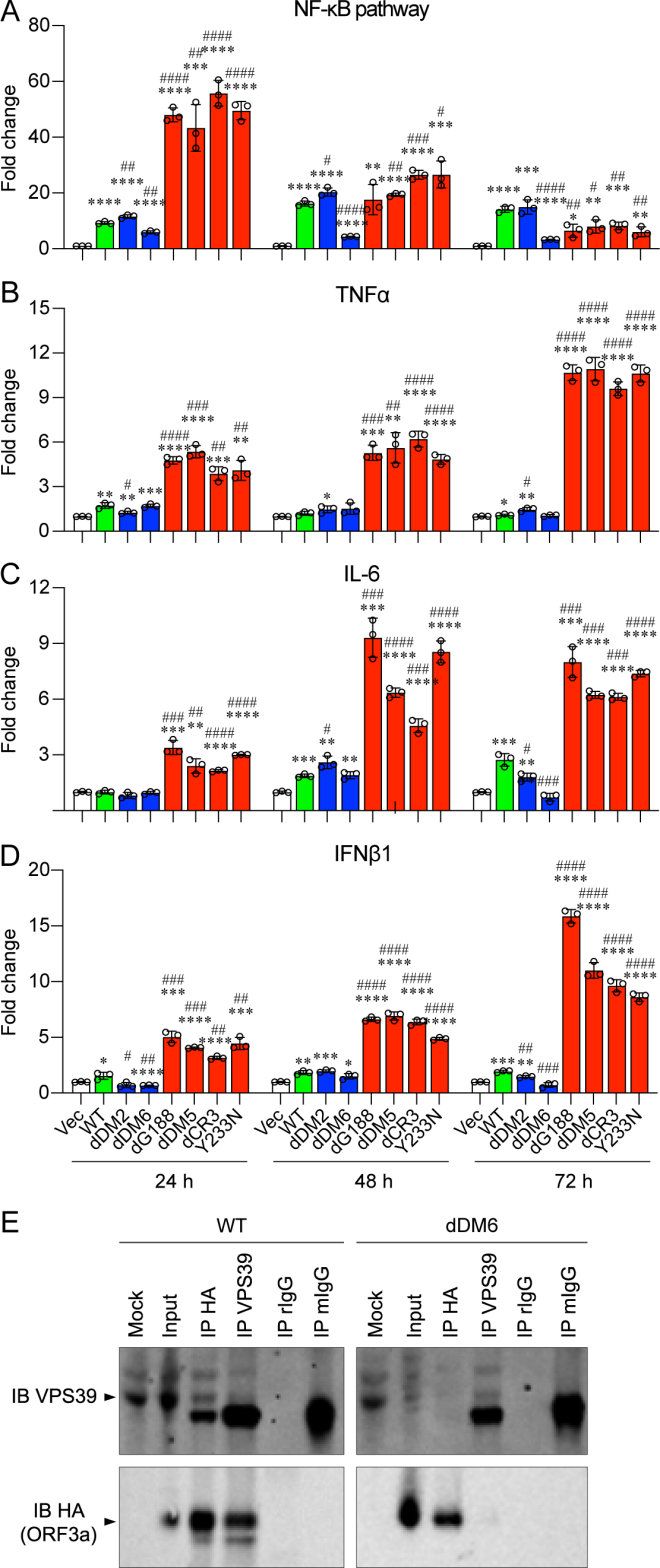
Elevated NF-kB and cytokine production in E-ORF3a proteins compared to L-ORF3a proteins. 293T cells were transfected with a pCAG plasmid expressing either L-ORF3a or E-ORF3a mutants. Cells were collected over a period of 72 hpt. (**A**) NF-kB luciferase (*luc*) reporter plasmid system (Stratagene, La Jolla, CA) was employed to quantify NF-kB-mediated transcriptional activities (termed NF-kB pathway). Transcription levels of NF-kB-mediated cytokines like TNFα (**B**), IL-6 (**C**), and IFNβ1 (**D**) were assessed using reverse transcription-quantitative PCR (RT-qPCR). mRNA levels of each gene target were tracked over the indicated time. (**E**) The dDM6:∆aa171-173 mutant carrying a deletion of aa171-173 in ORF3a fails to interact with VPS39 compared with the WT ORF3a. 293T cells were transfected with WT or dDM6:∆aa171-173 mutant ORF3a-carrying plasmid DNA. At 24 hpt, transfected cells were lysed for co-IP analyses. The pulled-down proteins were probed with anti-HA or anti-VSP39 antibody. rIgG and mIgG are negative controls and used to eliminate possible background signals generated by for rabbit and mouse antibodies used for IP. The plasmid used for measuring NF-kB-mediated pathways contains a firefly *luc* gene, driven by 5 copies NF-kB-binding and activating elements positioned upstream of the minimal TATA promoter. Upon activation by proinflammatory cytokines, endogenous NF-kB transcription factors bind to DNA response elements normally present in TNFα or IL-6 promoters, leading to transcription of the *luc* reporter gene. Consequently, Luc production signifies NF-kB pathway activation. Data are depicted as mean ± SE from three independent experiments. Statistical significance between WT ORF3a and mutant ORF3a (indicated by #) or between Vec and ORF3a (indicated by *) was assessed: * or #, *P* < 0.05; ** or ##, *P* < 0.01; *** or ###, *P* < 0.001; **** or ####, *P* < 0.0001 (one-way ANOVA), respectively. Arrows indicate the protein of interest.

Given our previous findings that ORF3a activates NF-kB (nuclear factor kappa B) and subsequent cytokine production (14), our next objective was to investigate whether L-ORF3a and E-ORF3a proteins differentially impact the host proinflammatory immune response. To commence, we monitored the activity of the transcription factor NF-kB over time. NF-kB plays a pivotal role as a master regulator in mediating cellular proinflammatory responses during viral infections (41). We utilized a NF-kB luciferase (Luc) reporter plasmid system (Stratagene, La Jolla, CA) to gauge NF-kB-mediated transcriptional activities. This plasmid contains a firefly luciferase gene driven by five copies of NF-κB-binding and activating element situated upstream of the minimal TATA promoter. Upon activation by ORF3a (14) or by a proinflammatory cytokine like TNFα, endogenous NF-κB binds to DNA response elements, resulting in the transcription of the *luc* reporter gene. Consequently, Luc production signifies the activation of the NF-kB pathways, which, in turn, triggers downstream cytokine production. As illustrated in Fig. 3A, the expression of E-ORF3a (in red bars) elicited a rapid and significantly higher level of NF-kB-mediated transcriptional activity than that of L-ORF3a proteins at 24 hpt. However, the difference in NF-kB activities between the two groups of proteins gradually diminished as the surge of NF-kB activity induced by the E-ORF3a gradually subsided over time.

Subsequently, we compared the production of the NF-kB-mediated cytokines including TNFα, IL-6, and type I interferon beta 1 (IFNβ1) between the two groups of ORF3a proteins through reverse transcription-quantitative PCR (RT-qPCR) (Fig. 3B through D). We observed an inverse relationship between NF-kB and the production of TNFα, IL-6, and IFNβ, which is consistent with the notion that the cytokine production of TNFα, IL-6, and IFNβ is regulated by NF-kB activation. Specifically, the transcriptional activities of all three cytokines induced by the E-ORF3a proteins (in red bars) increased over time following NF-kB activation, and the mRNA levels of these three cytokines induced by the E-ORF3a proteins were significantly higher than those induced by the L-ORF3a proteins. Conversely, the transcriptional levels induced by the L-ORF3a proteins were relatively low, with no clear trend of temporal changes. These data suggest that ORF3a proteins residing in the ER cause significantly higher levels of NF-kB-mediated cellular proinflammatory responses, resulting in elevated cytokine production, compared to those localized in lysosomes.

Among the L-ORF3a proteins tested, the dDM6:∆aa171-173 mutant exhibited a further reduction in cellular oxidative stress (Fig. 2) and proinflammatory immune responses (Fig. 3), resulting in additional attenuation of cell death. The dDM6:∆aa171-173 mutant encompasses a deletion of three amino acids from aa171-173. An early report demonstrated that a mutation at aa171 renders ORF3a unable to bind VPS39, a component of HOPS that involves in protein sorting and vesicle fusion (40, 42). Since HOPS bridges closely apposed autophagosomal and lysosomal membranes, mediating autophagosome–lysosome fusion (28), which is part of the host autophagic response (25), the interaction of ORF3a with VPS39 prevents the fusion of autophagosomes with lysosomes (29, 30). However, the functional consequences of losing the ORF3a-VPS interaction are not well understood. Here, we examined the interaction of dDM6:∆aa171-173 with VPS39 using co-IP analysis. As illustrated in Fig. 3E (left), as expected, the WT ORF3a protein was detected in the VPS39 pulldown products. Conversely, VPS39 was also detected in the HA-ORF3a pulldowns, confirming ORF3a binds to VPS39 as previously reported (29, 30, 40). In contrast, the dDM6 mutant does not bind VPS39, as neither the VPS39 nor the ORF3a pulldowns indicated an interaction between the dDM6:∆aa171-173 mutant and VPS39 (Fig. 3E, right). These data suggest that the reduced cellular oxidative stress and proinflammatory responses observed in the dDM6:∆aa171-173 mutant could potentially be attributed to the loss of its interaction with VPS39.

### Lysosomal-associated ORF3a proteins elicit strong reticulophagy response compared to ER-localized ORF3a proteins

**Fig 4:**
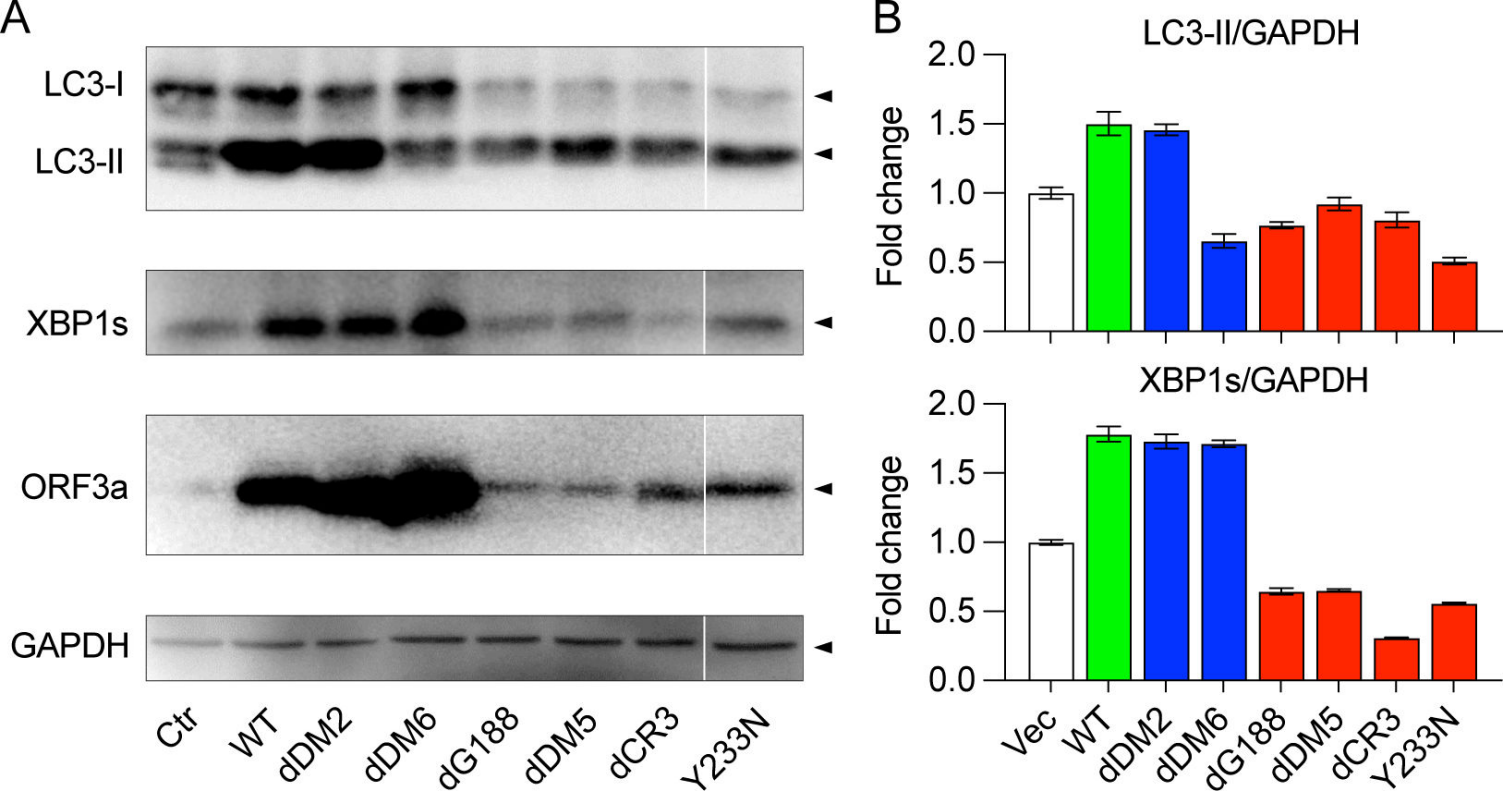
Enhanced cellular autophagy and ER stress responses observed in L-ORF3a compared to E-ORF3a proteins. 293T cells were transfected with a pCAG plasmid expressing either L-ORF3a or E-ORF3a mutant variants. Cells were collected, and cellular proteins were extracted at 24 hpt. (**A**) Western blot analysis results depict the induction of cellular autophagy and ER stress, determined by the protein level of LC3-II over baseline and the detection of XBP1s, using anti-LC3B and anti-XBP1s antibodies, respectively. The ORF3a protein level was detected using anti-ORF3a antibody, and glyceraldehyde-3-phosphate dehydrogenase (GAPDH) protein levels were assessed by anti-GAPDH antibody as protein-loading controls. Note that all information provided is from the same gel. The division between pCR3 and Y233N is intentional to indicate that other irrelevant lanes have been removed. (**B**) Protein levels of LC3-II and XBP1s identified through western blot analysis were quantified via densitometric analysis, as illustrated in the bar graph displaying mean ± SE from a minimum of three independent experiments.

The association between ORF3a and VPS39 is primarily driven by the induction of cellular autophagy (29, 30). Consequently, the disconnection between the dDM6:∆aa171-173 mutant and VPS39 results in, among the L-ORF3a proteins, a notable reduction in oxidative stress-mediated ROS production (Fig. 2E), diminished NF-kB-mediated cytokine production (Fig. 3), and a decrease in apoptotic cell death (Fig. 2C and D). These findings lend strong support to the concept that ORF3a-induced autophagy is intricately linked to the observed cytopathic effects. Here, we further conducted a comparative assessment of the capacities of L-ORF3a and E-ORF3a proteins to induce cellular autophagy responses (43, 44). In this experiment, we transfected 293T cells with a pCAG plasmid expressing either L-ORF3a or E-ORF3a mutant variants. At 24 hpt, we collected cellular protein extracts for Western blot analysis (43). The conversion of microtubule-associated protein light chain 3-I (LC3-I) to LC3-II serves as a conventional indicator of autophagy induction (43, 44). Since ORF3a not only triggers cellular autophagy but also prevents the fusion of autophagosomes with lysosomes (30, 45), the level of LC3-II protein can also be influenced by the abundance of autophagosomes (44). Therefore, we anticipated an additional increase in LC3-II. Thus, we used the accumulation of LC3-II as a collective reflection of ORF3a-induced autophagy activation and the concerted effort of ORF3a to block the fusion of autophagosomes with lysosomes (43, 44). It is also important to note that the level of LC3-I protein can also be used as a reference to monitor the conversion of LC3-I to LC3-II during autophagy activation. In the control cells transfected with the vector alone, we observed a relatively equal protein intensity between LC3-I and LC3-II, as depicted in Fig. 4A (top lane). In contrast, there was a significant augmentation in LC3-II protein intensity in cells expressing WT ORF3a and the dDM2:∆aa36-40 mutant, compared to the baseline observed in control cells. Notably, the dDM6:∆aa171-173 mutant, which lacks the ability to bind VPS39 (Fig. 3E) and, thus, is incapable of inducing autophagy, exhibited no accumulation of LC3-II protein. Intriguingly, little to no accumulation of LC3-II proteins was observed in cells expressing any of the four ER-residing ORF3a proteins, as illustrated in Fig. 4B (red bars). However, when the LC3-I levels of E-ORF3a is considered, they are clearly less than that of the vector control, suggesting E-ORF3a does not completely diminish autophagy.

Additionally, we assessed the levels of X-box-binding protein 1 (XBP1), a pivotal regulator of the unfolded protein response activated by IRE1α during ER stress (46). XBP1 activation involves a gene-splicing event that generates the mature XBP1 protein (XBP1s) (47). Since ORF3a is known to induce ER stress by upregulating XBP1s (15, 48), here, we merely chose it as a marker and specifically quantified XBP1s’ levels as an indicator of ORF3a-induced ER stress. As depicted in Fig. 4A (second lane), the levels of XBP1s proteins were notably higher in cells expressing all three L-ORF3a proteins compared to their E-ORF3a counterparts. Remarkably, XBP1s protein levels in cells producing E-ORF3a variants were comparable to those in the vector control cells, suggesting that E-ORF3a did not elevate ER stress at least at the time of the detection. Furthermore, we quantified the XBP1s protein levels using densitometric analysis normalized against the housekeeping protein glyceraldehyde-3-phosphate dehydrogenase (GAPDH). None of the XBP1s protein levels in cells expressing E-ORF3a variants exceeded those in control cells, as illustrated in Fig. 4B. It is noteworthy, however, that the levels of ORF3a protein residing in the ER were significantly lower than those associated with lysosomes (Fig. 4A, third lane). This discrepancy is addressed in the following experiment.

### The diminished ER stress and autophagy response observed with E-ORF3a proteins are likely attributed to ER-associated TRIM59 and 26S proteasome-mediated degradation of ORF3a protein

**Fig 5:**
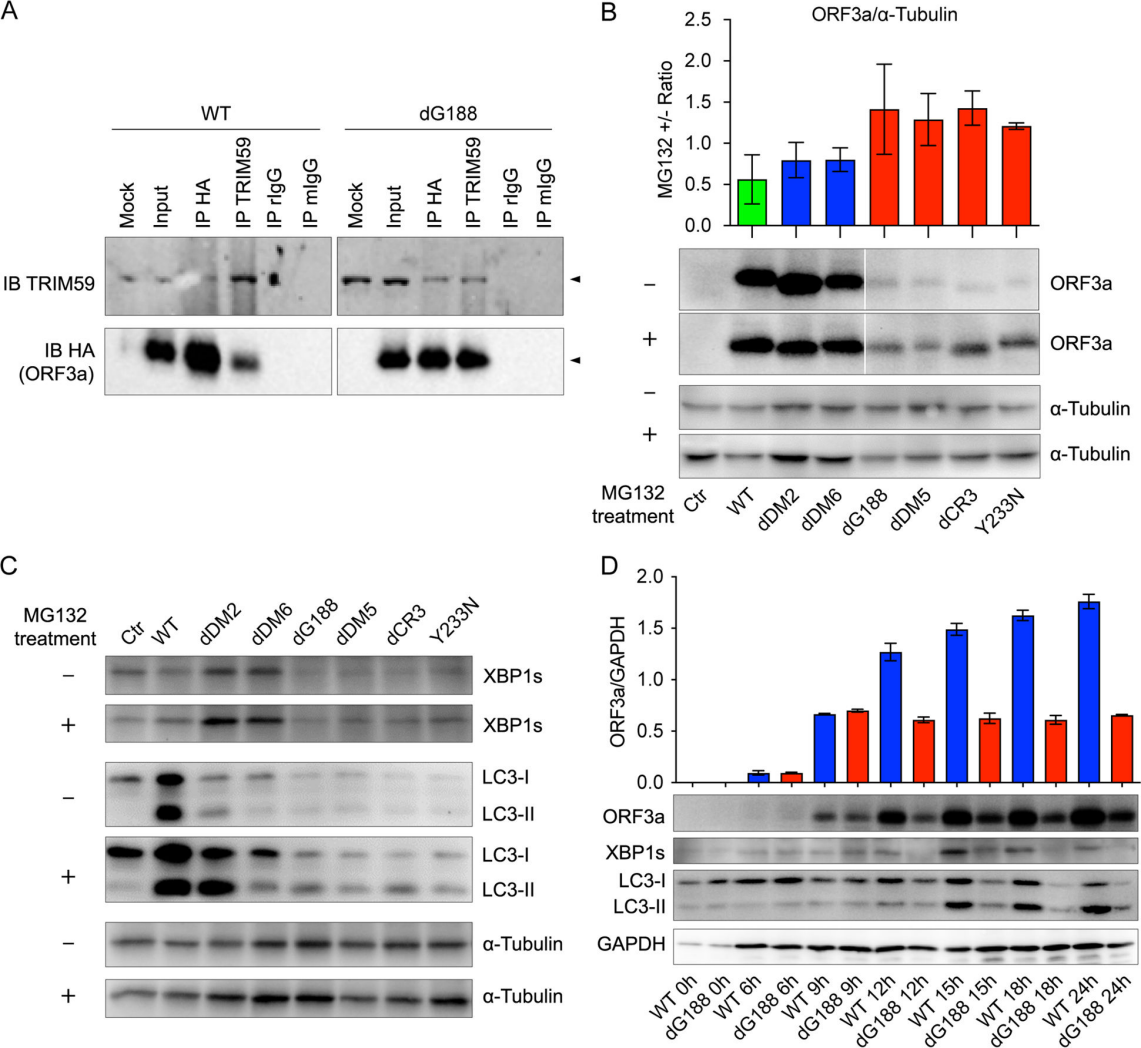
Impaired autophagy and ER stress response in the ER is likely due to ER-associated 26S proteasome-mediated ORF3a protein degradation. 293T cells were transfected with a pCAG plasmid expressing either L-ORF3a or E-ORF3a mutants. Cells were collected, and cellular proteins were extracted at 24 hpt. (**A**) Reciprocal co-IP unveiled the interaction of WT or dG188 ORF3a protein with the E3 ligase TRIM59. Comparative densitometric analysis of the pull-down ORF3a proteins indicates the dG188 protein is about 1.5-fold higher in ER than the WT in the lysosomes. (**B**) Treatment of ORF3a-producing cells with a 26S proteasome inhibitor, MG132, led to partial restoration of E-ORF3a protein levels. Changes in ORF3a protein levels were measured by the ratio protein intensities between treated vs untreated cells out of four independent Western blots. The accompanied escalation in the autophagy and ER stress responses were measured and shown in panel **C**. (**D**) Early time-course monitoring of L-ORF3a and E-ORF3a proteins indicates that little or no protein degradation was seen in the lysosomal-associated ORF3a proteins. In comparison, the levels of E-ORF3a proteins were comparable with the L-ORF3a proteins up to 9 hpt; thereafter, they started to degrade presumably due to ERAD. For the MG132 treatment experiment, 2 µM of MG132 (Selleckchem, Lot B38878) was added to transfected 293T cells at 16 hpt. The cells were harvested at 24 hpt (i.e., 8 h post-MG132 treatment). Protein samples were extracted and subjected to western blot analysis, employing the same antibodies as used in Fig. 4. Protein levels, as determined through western blot analysis (**B and D**), were quantified by densitometric analysis. The results are depicted in the bar graph, presenting mean ± SE from a minimum of three independent experiments.

Our data shown in Fig. 4 strongly suggest the potential involvement of ERAD via 26S proteasome-mediated protein degradation for ORF3a proteins localized within the ER. The process of ERAD is known to be a quality control mechanism that specifically target misfolded ER proteins for ubiquitination and subsequent degradation (31), and thus, it may provide an explanation for the observed differences in ORF3a protein levels and their associated responses. Consistent with previous studies (46–48) demonstrating the association of WT ORF3a protein with TRIM59, our experiments confirmed this interaction (Fig. 5A, left). Moreover, we found that the dG188 mutant, which represents an ER-localized ORF3a protein, also co-precipitated with TRIM59 (Fig. 5A, right). Comparative densitometric analysis of the pull-down ORF3a proteins indicates that the dG188 protein is about 1.5-fold higher in ER than the WT in the lysosomes. This observed association further supports the hypothesis of 26S proteasome-mediated protein degradation of ORF3a through TRIM59-mediated ubiquitination.

Next, we tested whether MG132, a 26S proteasome inhibitor, can reverse E-ORF3a protein levels in transfected 293T cells. MG132 treatment was initiated at 16 hpt, and cells were harvested at 24 hpt (8 h post-MG132 treatment). Protein samples were extracted and subjected to Western blot analysis, allowing us to assess the relative levels of ORF3a proteins compared to untreated controls. Changes of ORF3a protein levels were measured by the ratio protein intensities between treated vs untreated cells. As illustrated in Fig. 5B, the L-ORF3a proteins showed no significant change in protein levels upon MG132 treatment. In contrast, MG132 treatment led to a partial restoration of E-ORF3a protein levels, resulting in an approximately 1.2- to 1.4-fold increase (Fig. 5B).

To investigate whether the restored E-ORF3a proteins could induce cellular autophagy and ER stress responses, we employed the same methods used in Fig. 4 to detect autophagy and ER stress. In comparison to the untreated controls, a slight increase in the levels of LC3-II and XBP1s proteins was observed (Fig. 5C). This suggests that ER-associated ORF3a proteins are able to trigger cellular autophagy and ER stress responses, possibly through an interplay with the reticulophagy response (31).

To address the discrepancy regarding why E-ORF3a proteins induce heightened cytopathic effects despite their lower levels of ORF3a proteins and ER stress, we conducted a time-course experiment to monitor and compare the levels between the WT and dG188 proteins immediately after the introduction of ORF3a-carrying plasmids into the cells. As shown in Fig. 5D, the WT and dG188 proteins were produced as early as 6 hpt, and their protein levels remained comparable until 9 hpt when the dG188 mutant also induces ER stress. Thereafter, the dG188 protein levels started to decay and reduced to the levels that are less than the WT, presumably due to ERAD.

## DISCUSSION

In our early comprehensive mutagenesis study (5), which involved a range of artificially engineered ORF3a mutant proteins, we identified two distinct categories of ORF3a proteins based on their subcellular localization: those resembling the WT ORF3a and primarily residing on the lysosomal membrane (L-ORF3a), and those predominantly found within the endoplasmic reticulum (E-ORF3a) (5). In this study, with a selected panel of ORF3a mutants (Fig. 1A), we further confirmed in a different and renal epithelial HK2 cell line that we can distinguish these two types of ORF3a mutant proteins from the WT ORF3a based on their subcellular localizations (Fig. 1B). Additionally, we demonstrate that ORF3a proteins localize on the lysosomal or ER membranes by binding to LAMP-1 or Calnexin, respectively (Fig. 1C). These observations are not surprising as ORF3a, as a membrane-associated protein, undergoes a series of intracellular trafficking events following its synthesis in the ER (20) and subsequent processing in the Golgi complex (21, 22). This process leads to the distribution of ORF3a across plasma membrane (23, 49, 50) and various endomembranes, including lysosomes (29, 51–53). Therefore, the movement of ORF3a within the cell is dynamic, and its presence can be detected in both the ER and lysosomes at any given time point of detection (Fig. 1B–a and b). However, the key distinction we observed from normal intracellular transport is that, due to specific mutations in the ORF3a protein, the mutant variants either become trapped within the ER or experience delayed release. It is important to note that our experimental approach, which involved analyzing cells at a fixed time point, does not allow us to differentiate between complete entrapment and temporal delays in ER release. To further substantiate the lysosomal- or ER-associated ORF3a effects as described here, in the future, we would consider fusing an ER-specific or lysosome-specific targeting signal sequence to ORF3a and direct it specifically to either ER or lysosomes. However, the potential challenge is that, unless we can knowingly eliminate indigenous ER or lysosome-homing sequences, it might further complicate interpretation of the observed results.

Early mutational analyses, including our own, have identified specific functional domains within the ORF3a protein, such as the YXXΦ motif (aa160-163) and the diG motif (aa187-188) in the cytoplasmic region, which play crucial roles in directing ORF3a’s intracellular transport from the ER to lysosomes (5, 23, 24). Consequently, any mutations that impact the intracellular transport of ORF3a are likely to result in the retention of the protein within the ER. Consistent with this notion, we demonstrated that mutations in the diG motif, such as the dG188 mutant, lead to ER retention (Fig. 1B). Interestingly, several naturally occurring ORF3a mutant variants (G188C/D/V) were found to affect the G188 residue, which is structurally vital and critical for the intracellular transport of ORF3a (1, 5, 6, 14). Similarly, the dDM5:∆aa160-163 mutant, deleting the YXXΦ motif, also localizes primarily to the ER. The reason for the dCR3:∆aa215-234 mutant’s retention in the ER remains unclear. However, an *in silico* analysis of the ORF3a structure suggests that the Y233 residue might constitute another potential YXXΦ motif (aa233-236: YNKI) (54). It is also interesting to note that the dCR3:∆aa215-234 mutant encompasses a region that includes naturally occurring mutations such as Y233N and T223I, with the latter being identified in the Omicron variants (1). Nevertheless, the functional consequences of these functionally defective or naturally occurring ORF3a mutant proteins, which are retained in the ER, and how they differ from those that proceed to lysosomes, have remained largely unknown.

While both L-ORF3a and E-ORF3a proteins have been shown to induce cytopathic effects by increasing NF-kB-mediated cytokine levels (14, 15, 17, 27), the precise molecular mechanism responsible for NF-kB activation by ORF3a remains elusive. Initial reports suggested that the interaction of TRAF3 with ORF3a is necessary for ORF3a-induced NF-κB and NLRP3 inflammasome activation (10, 39). However, our data indicate that the deletion of the TRAF3-binding site in the dDM2:∆aa36-40 mutant does not have a clear effect on NF-kB activation or NF-kB-mediated cytokine production (Fig. 3). The cause of this discrepancy is presently unclear. One possible explanation may be attributed to the use of different cell lines. Another study has proposed that ORF3a activates NF-kB by facilitating the interaction between IKKβ and NEMO, an essential step in NF-kB activation (55). This activation is achieved through direct interactions between ORF3a and both IKKβ and NEMO (17). Indeed, our IFA has indicated that NF-kB protein translocates into the nucleus shortly after ORF3a protein production, which is a post-step of NF-kB activation by IKKβ and NEMO (data not shown). However, our earlier research has hinted potential differences in how NF-kB is activated by L-ORF3a and E-ORF3a (14). Specifically, we found that L-ORF3a, like the WT ORF3a, activates TLR3, while E-ORF3a with the dG188 mutation activates both TLR3 and TLR4 (14). It is worth noting that TLR3 and TLR4 may exhibit distinct preferences in their localization, residing either in endosomal compartments or on the plasma membrane (56–58). Importantly, both TLR3 and TLR4 are known to recognize RNA viruses and trigger NF-kB-mediated proinflammatory responses, contributing to the severity of COVID-19 (59–61). As a result, we hypothesize that the preferential activation of TLR3 and/or TLR4 by L-ORF3a or E-ORF3a could lead to discernible differences in the induction of NF-kB activation. We plan to test this hypothesis in our future experiments.

Our investigation on the dDM6:∆aa171-173 mutant has established a direct connection between NF-kB-mediated cytokine production, cytopathic effects, and cellular autophagy responses induced by L-ORF3a proteins. While we have demonstrated that the dDM6:∆aa171-173 mutant leads to reduced NF-kB-mediated cytokine production (Fig. 3) and cytopathic effects compared to the wild type (Fig. 2), this mutation also fails to bind VPS39 (Fig. 3E) and induce autophagy (Fig. 4). These findings strongly suggest that ORF3a-induced autophagy contributes, at least in part, to the observed cytopathic effects. Notably, the interplay between autophagy and NF-κB signaling pathways has been extensively documented (62). However, the precise mechanisms governing the interaction between NF-kB and autophagy during the induction of cytopathic effects by ORF3a remain unclear.

Furthermore, our results have revealed a significant crosstalk between ER stress and autophagy, a process referred to as reticulophagy or ER-phagy (31). We compared the induction of autophagy and ER stress, as assessed by the accumulation of LC3-II and the levels of XBP1s (27, 43, 44). As anticipated, ORF3a proteins primarily localized to lysosomes not only induce autophagy but also trigger ER stress (Fig. 4) (48). An interesting exception is observed with the dDM6:∆aa171-173 mutant, which is unable to induce autophagy but still induces ER stress (Fig. 4). Given that it is unable to associate with VPS39 of the HOPS complex, components of the cellular antiviral autophagy response (Fig. 3E) (40, 42), it is reasonable to assume that the mutant has lost its ability to induce reticulophagy while retaining its capacity to trigger ER stress. However, the impact of this mutant’s effects on the interplay between ORF3a-induced ER stress and autophagy remains elusive.

The ORF3a-induced cellular reticulophagy response has been previously documented (25, 27) and reviewed by others (26). Mechanistically, ORF3a triggers RETREG1/FAM134B-related reticulophagy through the high mobility group box 1 (HMGB1)-Beclin 1 pathway (25). RETREG1/FAM134B serves as a reticulophagy receptor responsible for regulating ER turnover (63). The association of HMGB1, an extracellular damage-associated molecular pattern molecule, with Beclin 1, a critical autophagic protein, is instrumental in initiating reticulophagy (64). It has been proposed that during SARS-CoV-2 infection, as ORF3a localizes to the ER, it interacts with HMGB1, consequently enhancing the association between HMGB1 and Beclin 1 to initiate RETREG1-mediated reticulophagy. This, in turn, leads to the induction of ER stress by ORF3a, thereby facilitating SARS-CoV-2 infection, triggering proinflammatory responses, and promoting Caspase-12-mediated and ER-specific apoptosis (25).

Unexpectedly, despite the more pronounced cytopathic effects observed with ORF3a proteins localized in the ER compared to those associated with lysosomes, the E-ORF3a proteins triggered barely detectable levels of ER stress or autophagy (Fig. 4). We hypothesize that this could potentially be attributed to ERAD activated by the cellular reticulophagy response. ERAD is known to play a crucial role in the timely removal of ER-damaged proteins, thereby protecting cells from the harmful effects of excessive ER stress (65). Since ERAD is a quality control mechanism that targets specifically ER-residing proteins, this could potentially explain as why L-ORF3a proteins are more resistant to ERAD. Consistent with this notion, we observed a significant reduction in the protein levels of the E-ORF3a when compared to the L-ORF3a proteins (Fig. 4, third lane). Additionally, previous reports have indicated that WT ORF3a binds to a Ub E3 ligase known as TRIM59 (66–68). In our study, we have extended these findings by demonstrating that the ER-localized dG188 mutant also associates with TRIM59 (Fig. 5A). Notably, TRIM59 is a Ub E3 ligase primarily located in the ER (69), further supporting the concept of ER-associated and 26S proteasome-mediated degradation of ORF3a proteins. Our suspicions regarding this process were confirmed when we treated cells with MG132, a 26S proteasome inhibitor, which resulted in a partial restoration of E-ORF3a protein levels, whereas little or no change was seen in the L-ORF3a proteins (Fig. 5B). Interestingly, in line with the mild increase in ORF3a protein levels following MG132 treatment (Fig. 5B), there was a notable rise in the accumulated LC3-II and XBP1s proteins (Fig. 5C). This suggests a reverse correlation between ORF3a protein degradation and the activation of autophagy and ER stress responses. However, there is a conflicting observation. Despite the expectation that E-ORF3a protein should exclusively bind to TRIM59 in the ER, a substantial amount of WT ORF3a protein also binds to TRIM59 (Fig. 5A). One possible explanation for this could be that some of the WT proteins remained in the ER at the time of our test, as indicated by the ImageJ analysis (Fig. 1A–b). Alternatively, it is plausible that TRIM59 is not solely confined to the ER but may also be present in lysosomes.

There is also another discrepancy as why the MG132 treatment did not completely restore the levels of E-ORF3a protein in contrast to its complete restoration effect on other proteins in our previous research (70). We suspect that the partial restoration of the E-ORF3a proteins we observed may be attributed to either the high levels of ORF3a proteins or the late time point we analyzed. In other words, can the presence of a low level of ORF3a protein in the ER lead to such a remarkably high level of cytopathic effect (Fig. 2)? One plausible explanation for this discrepancy is that as soon as an E-ORF3a protein is synthesized in the ER, it triggers a significantly more pronounced cytopathic effect via ER stress than those localized to lysosomes even though they will ultimately be degraded by ERAD. This premise is somewhat supported by our time-course monitoring of E-ORF3a proteins, where the levels of E-ORF3a protein are comparable to those of L-ORF3a proteins at early time points such as 9 hpt when the dG188 mutant also induces a small increase of XBP1s (Fig. 5D). These early-produced E-ORF3a proteins could potentially be sufficient to induce the observed potent cytopathic damage. However, this possibility is less likely as L-ORF3a proteins are also exported from ER at early time point. Alternatively, it is possible that the ER, being highly sensitive to foreign proteins, responds strongly even to small amounts of E-ORF3a that are retained in the ER, leading to significant cytopathic effects.

**Fig 6:**
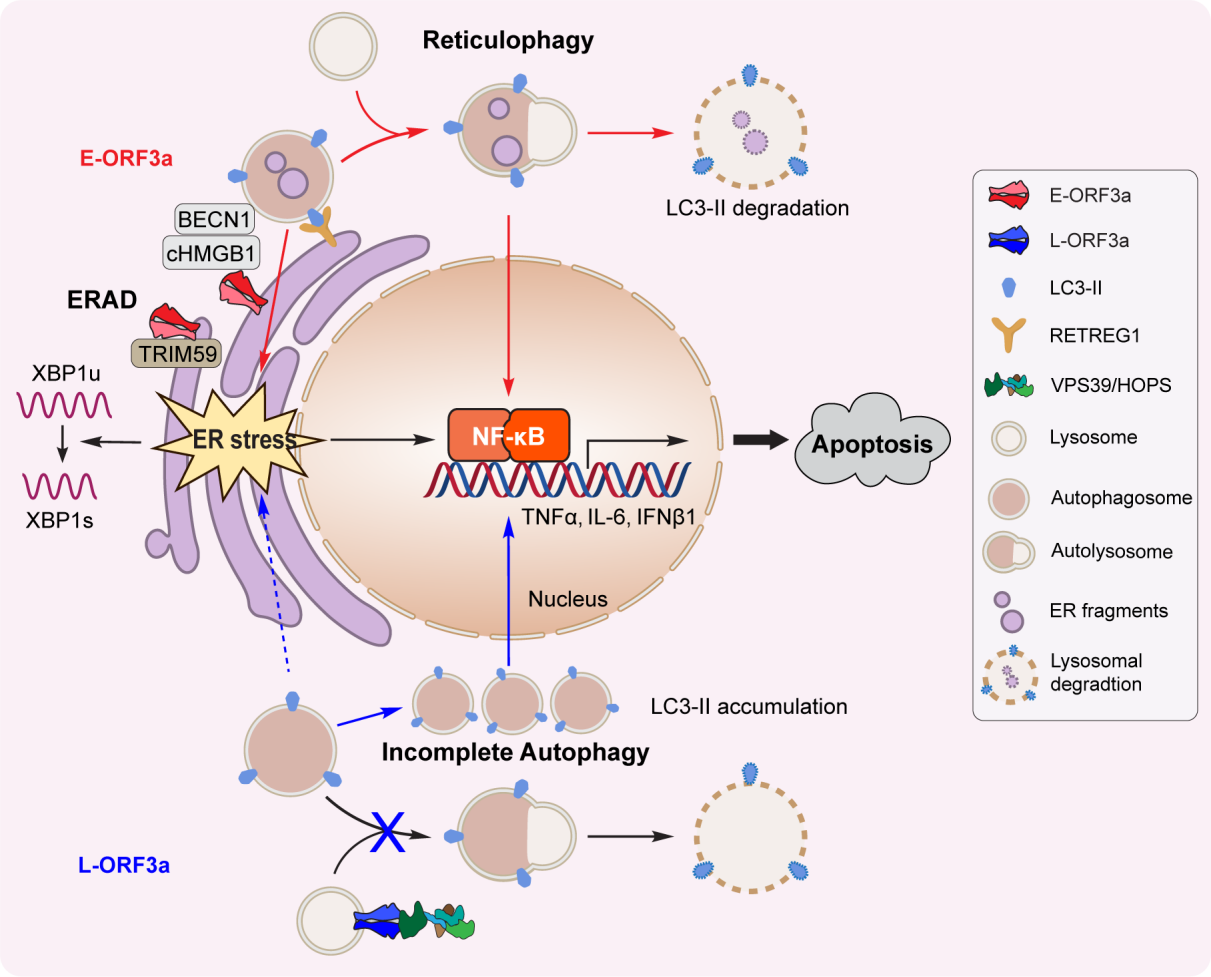
A working model outlining the actions of the ORF3a protein within the ER and lysosomes. When ORF3a localizes within the ER, it initiates RETREG1/FAM134B-related reticulophagy through the HMGB1-Beclin 1 pathway (25). This, in turn, induces ER stress, partially through the activation of IRE1α-mediated XBP1s production. Subsequently, this cascade leads to NF-kB activation and the transcription of TNFα, IL-6, and IFNβ1, ultimately culminating in apoptosis. Furthermore, the activation of reticulophagy within the ER triggers TRIM59 E3 ligase-mediated protein ubiquitination and subsequent ERAD of E-ORF3a via 26S proteasome-mediated proteolysis. On the other hand, when ORF3a localizes to the lysosomal membrane, it induces a cellular autophagy response. However, it concurrently inhibits the fusion of autophagosomes with lysosomes by interacting with the VPS39/HOPS complex. This obstruction also results in NF-kB activation and NF-k-mediated cytokine production, ultimately leading to apoptosis. Due to the sensitivity of the ER, E-ORF3a proteins exert more profound cytopathic effects than their lysosomal counterparts despite the robust ER-associated degradation.

Based on the findings described in this report, we have formulated a working model regarding the actions of the ORF3a protein, which is found in both the ER and lysosomes (Fig. 6). Specifically, when ORF3a localizes in the ER, it triggers RETREG1/FAM134B-related reticulophagy via the HMGB1-Beclin 1 pathway (25), which, in turn, induces ER stress, partially through the activation of IRE1α-mediated XBP1s production. Subsequently, it leads to NF-kB activation and subsequent production of proinflammatory cytokines (TNFα and IL-6) and type 1 IFNβ1, ultimately resulting in apoptotic cell death. Furthermore, the activation of reticulophagy in the ER also instigates TRIM59 E3 ligase-mediated protein ubiquitination and the subsequent degradation of E-ORF3a via the 26S proteasome. As ORF3a progresses and localizes to the lysosomal membrane, it triggers a cellular autophagy response. However, it simultaneously blocks the fusion of autophagosomes with lysosomes by interacting with VPS39, a component of the HOPS complex responsible for mediating this fusion. This obstruction also leads to NF-kB-mediated cytokine production, resulting in apoptosis. Altogether, ORF3a proteins, whether situated in the ER or lysosomes, induce apoptosis by eliciting cellular oxidative stress and proinflammatory cytokine production. Nevertheless, due to the delicate nature of the ER, ER-associated ORF3a proteins exert more profound cytopathic effects than their lysosomal counterparts despite robust ER-associated degradation.

Given that ER-residing ORF3a proteins are found in naturally occurring ORF3a mutants, our results raise the possibility that emerging SARS-CoV-2 variants carrying certain ORF3a mutations may exhibit distinct cytopathic effects on host cells. This highlights the potential significance of ORF3a in the pathogenicity of SARS-CoV-2 variants and warrants further investigation into the implications of these findings for viral virulence and host responses.

## MATERIALS AND METHODS

### Cell lines, growth media, and plasmid transfection

Two human cell lines were used for this study: the human renal proximal tubule epithelial cell line HK2 (ATCC CRL-2190) and the human embryonic epithelial cell line HEK293T (ATCC CRL-1573). The HK2 cell line is typically cultivated using a specialized medium known as Keratinocyte Serum-Free Medium (K-SFM; Life Technologies Cat#: 17005-042). However, both cell lines can be sustained in Dulbecco’s modified Eagle’s medium (DMEM), supplemented with 10% fetal calf serum (FCS), penicillin (100 IU/mL), streptomycin (100 µg/mL), and amphotericin B (2.5 µg/mL). For the immunofluorescent assays, we discovered that a 1:1 mixture of K-SFM and DMEM yields superior results for HK2 cells, as it promotes their superior adherence to the culture petri dish.

To investigate the functions and subcellular localization of both the WT and ORF3a mutant proteins, pCAG plasmid carrying gene of interest was transfected into 293T or HK2 cells using Lipofectamine 3000 following the manufacture protocol (Thermo Fisher Scientific).

### Immunofluorescent assay and confocal microscopy

For IFA, cells were cultivated on coverslips and subsequently fixed with 1% paraformaldehyde for a duration of 10 min at room temperature. Following fixation, the cells were permeabilized with 0.2% Triton-X100 for 20 min on ice. Subsequently, a sequential series of incubations was performed. The cells were first incubated with primary antibodies, followed by incubation with Texas Red (TR)- or FITC-labeled secondary antibodies, all of which were dissolved in phosphate-buffered saline (PBS). Each incubation step lasted for 30 min. Finally, the cells were equilibrated in PBS, stained to visualize DNA using Hoechst 33258 at a concentration of 0.5 µg/mL, and then securely mounted using Fluoromount G (Fisher Scientific, Newark, DE).

We utilized a Leica TCS SPII confocal laser scanning system to examine the cells. During imaging, we recorded two or three channels either simultaneously or sequentially. We took special care to control to minimize potential signal interference between the fluorescein isothiocyanate and Texas Red channels, as well as between the blue and red channels.

### Quantification of ORF3a co-localization with lysosomes or ER

The quantification method for assessing the co-localization of ORF3a with different organelles has been described previously (5). In brief, we employed the ImageJ2 image software in conjunction with the JACoP plugin (https://imagej.net) (36, 37). First, we took a merged image of selected ORF3a-positive cells and split it into its red and green channels using the color function within the image menu. Subsequently, we utilized the JACoP plugin to analyze the co-localization of the proteins of interest. We maintained the default threshold for the red channel, which represented the markers of lysosomal or ER. For consistency, we set the threshold for the green channel (ORF3a) at 50%. After the analysis, we obtained both the Pearson’s correlation coefficient (*P*) and Mander’s overlap coefficient (*M*) to assess the degree of co-localization between ORF3a and the organelle biomarker proteins. We conducted this analysis by examining at least 10 random images and calculated the mean and standard deviation for both *P* and *M* values. Statistical significance was assessed using a pairwise Student’s *t*-test, with levels of significance indicated as follows: *P < 0.05, **P < 0.01, ***P < 0.001, and ****P < 0.0001, denoting various degrees of significance.

### Co-immunoprecipitation assay and immunoblot analysis

The co-immunoprecipitation assay and the subsequent immunoblot analysis were carried out following a standardized procedure. In brief, *ORF3a*-expresisng plasmid was transfected into 293T cells. At 20 hpt, cells were lysed by utilizing an ice-cold lysis buffer comprising 25 mM Tris-HCl (pH 7.4), 150 mM NaCl, 1% NP-40, 1 mM EDTA, and 5% glycerol. Additionally, this lysis buffer was supplemented with a protease inhibitor cocktail (Sigma, Cat# P8340), and the cell lysis process was performed on ice for a duration of 10 min. Following this step, the lysates underwent centrifugation at 3,000 *× g* for 5 min, and the resulting supernatants were transferred to new tubes.

For the subsequent immunoprecipitation, these supernatants were subjected to overnight incubation at 4°C in the presence of specific antibodies such as anti-HA, anti-Calnexin, anti-LAMP-1, anti-VPS39, and anti-TRIM59 (for detailed information of these antibodies, see Table S1) or normal mouse or rabbit IgG as a negative control. These incubation mixtures were then combined with protein G-Sepharose beads (Amersham Pharmacia Biotech AB, Sweden), as per the manufacturer’s instructions and allowed to incubate for an additional 3 h.

Subsequent to the immunoprecipitation, the beads underwent a triple wash with PBS containing 0.1% bovine serum albumin, along with a protease inhibitor cocktail. The immunoprecipitated complexes were then resuspended in a mixture of PBS and 2× Laemmli buffer (20 µL each). After being heated at 95°C for 5 min, the beads were separated by centrifugation, and the resultant supernatants were subjected to SDS-PAGE immunoblotting.

For the immunoblot analysis, protein samples (10–20 µg per lane) were separated utilizing a 7.5% polyacrylamide gel, followed by their transfer onto nitrocellulose membranes (Amersham Inc., Piscataway, NJ). Subsequently, these membranes were blocked with 5% nonfat milk for a period of 60 min at room temperature. Thereafter, the membranes were incubated overnight at 4°C with primary antibodies. Post-primary antibody incubation, the membranes were treated with a horseradish peroxidase-coupled secondary antibody (Amersham Inc.). Protein detection was achieved through an enhanced chemiluminescence method (Pierce, Rockford, IL), following standard protocols. To facilitate the detection of additional proteins of interest, the membranes were stripped using a stripping buffer (comprising 100 mM β-mercaptoethanol, 2% SDS, and 62.5 mM Tris-HCl, pH 6.8), washed with PBS containing 0.1% Tween 20, and subsequently reused for different protein detection.

### Measurement of cell growth, viability, and apoptotic cell death

293T cells were initially seeded into individual wells of a 96-well plate, with each well containing 2.4 × 10^4^ cells. These cells were left to incubate overnight at 37°C with a 5% CO_2_ atmosphere to ensure proper cell adherence and adaptation. Subsequently, we introduced 100 ng of plasmid DNA into these cells using the Lipofectamine 3000 reagent, following the manufacturer’s prescribed procedure. At 48 hpt, we first quantified cell growth and cell death by cell counting, involving trypsinization, the creation of a mixture with an equal volume of cell suspension and trypan blue staining solution, and cell counting using a TC20 automated cell counter (BioRad) to determine both the total cell count and the number of cells that had experienced cell death by trypan blue. Additionally, to assess cell viability, we conducted the MTT assay. This assay involved adding 10 µL of a 5 mg/mL MTT solution to each well of the 96-well plate and incubating it at 37°C for 2–5 h. After removing the medium, we added 100 µL of dimethyl sulfoxide (DMSO) to each well, gently mixed it, and further incubated the plates at 37°C for 15 min. Finally, luminescence (RLU) and fluorescence (RFU, 485 nmEx/520–530 nmEm) were used to measure apoptosis and necrosis, respectively, with the H1M microplate reader (Agilent).

### Measurement of reactive oxygen species

The quantification of cellular oxidative stress activation in 293T cells was achieved through an ROS assay, utilizing a ROS detection cell-based assay kit supplied by Cayman Chemical (Cat# 601290), following the manufacturer’s prescribed guidelines. The resulting fluorescence from DHE staining was observed using a BZX fluorescence microscope from Keyence, and its intensity was measured using the Synergy H1 microplate reader provided by Agilent.

### NF-κB luciferase assay

To assess NF-κB-mediated transcriptional activities, an NF-κB luciferase assay was conducted in accordance with our previously published protocol (14). In brief, 2.4 × 10^4^ 293 T cells were cultured in individual wells of a white 96-well plate overnight. For the assay, co-transfection involved 0.05 µg of the plasmid of interest, 0.05 µg of pNF-κB-Luc, and 0.01 µg of pRL-SV40. To quantify luciferase activities, we employed a dual luciferase reporter assay system sourced from Promega (Cat# E1910) and the Synergy H1 microplate reader from Agilent, at the specified time intervals. Signal normalization was achieved by using Renilla luciferase as a reference, followed by the calculation of fold changes when compared to the control with an empty vector.

### Reverse transcription-quantitative PCR

The RT-qPCR was performed following the methodology as previously described (14). Briefly, 293T cells were cultivated in a 6-well plate until a confluency of approximately 70% was reached. The cells were transfected with 2.5 µg of plasmid DNA. At 24, 48, and 72 hpt, the cells were harvested, and RNA was extracted using Trizol (Invitrogen, 448706). The extracted RNA sample was first treated with RQ1 RNase-Free DNase (Promega, M6101) in order to remove contaminating genomic DNA. The remaining RNA was then transcribed into cDNA by using Reverse Transcriptase (Thermo Fisher, 4311235). Real-time PCR was carried out using a QuantStudio 3 Real-time PCR system with gene-specific primers. SYBR Green Master Mix (Thermo Fisher, A46109) was employed to detect gene mRNA expression. The amplification conditions consisted of 40 cycles of 95°C for 10 s and 60°C for 30 s, followed by a melting curve analysis. The fold change in mRNA expression was quantified by calculating the 2^−ΔΔ*CT*^ value, with *GAPDH* mRNA utilized as an endogenous control.

### MG132 assay and analysis

For the MG132 treatment experiment, 293T cells were cultivated in a 6-well plate until they achieved 70% confluency. Subsequently, the cells were transfected with 2.5 µg of plasmid DNA. At 16 hpt, 2 µM of MG132 (Selleckchem, Lot B38878) was introduced. Cell harvesting was conducted at 24 hpt, and protein samples were extracted for subsequent Western blot analysis, which, as previously detailed (14), involved lysing the cells of interest on ice for 30 min using RIPA buffer, which contained PMSF and a protein inhibitor. Electrophoresis was conducted at 80–120 V for 1.5 h, and the resulting proteins were transferred onto a PVDF membrane (Bio-rad) at 300 mA for 40 min. Blocking was carried out using 5% bovine serum albumin for 1 h, followed by an overnight incubation with the primary antibody at 4°C. The membrane was washed three times with Tris-buffered saline with 0.1% Tween 20 detergent (TBST) for 5 min each, and subsequently, it was incubated with the secondary antibody at room temperature for 1 h. Further washing was performed three times with TBST. The protein of interest was detected as chemiluminescent signal by using the SuperSignal West Femto Maximum Sensitivity Substrate (BiologyThermo scientific, 34095) on the ChemiDoc Touch Imaging System (BioRad). The antibodies used in this study are summarized in Table S1.

### Statistical analysis

Pair-wise *t*-tests, one-way or two-way ANOVA was performed using Prism 9 software (GraphPad, San Diego, CA, USA). Statistical significance was considered at the 95% confidence level (*P* < 0.05). Symbols * and #, ** and ##, *** and ###, or **** and #### indicated levels of significance: *P* < 0.05, *P* < 0.01, *P* < 0.001, or *P* < 0.0001, respectively.

## SUPPLEMENTAL MATERIAL

Table S1 mbio.03030-23-s0001.docx Antibodies used in the study.

ASM does not own the copyrights to Supplemental Material that may be linked to, or accessed through, an article. The authors have granted ASM a non-exclusive, world-wide license to publish the Supplemental Material files. Please contact the corresponding author directly for reuse.
