# Supplementary material for: Endoplasmic reticulum-associated SARS-CoV-2 ORF3a elicits heightened cytopathic effects despite robust ER-associated degradation
Source: mBio. 2023 Dec 11;15(1):e03030-23. doi: 10.1128/mbio.03030-23 (PMC10790703; doi:10.1128/mbio.03030-23)
Supplement: Table S1 — Antibodies used in the study. [file mbio.03030-23-s0001.docx]

**Supplemental Materials**

**Table S1**. List of primary antibodies used in this study.

| **Antibody** | **Detection** | **Vendor / Cat #** | **Purpose (Dilution)** | **Species & Clonality** |
| --- | --- | --- | --- | --- |
| anti-αTubulin 4G1 | A house-keep protein | Santa Cruz, sc-58666 | WB | Mouse mAb |
| anti-αTubulin | A house-keep protein | Cell Signaling, 2144 | WB | Rabbit mAb |
| anti-Calnexin | ER | Abcam, ab22595 | IFA/IP | Rabbit pAb |
| anti-GAPDH | A house-keep protein | Sigma-Aldrich, MAB374 | WB | Mouse mAb |
| anti-HA | HA tag | Sigma, H6908 | IFA | Rabbit Ab |
| anti-LAMP-1 E-5 | Lysosomal membrane | Santa Cruz, sc-18821 | IFA/IP | Mouse mAb |
| anti-LC3B | Autophagy | Novus, NB100-2220 | WB | Rabbit pAb |
| anti-ORF3a | SARS-CoV-2 ORF3a | LSBio, C829863 | IFA/IP | Rabbit pAb |
| anti-ORF3a | SARS-CoV-2 ORF3a | RnDSystems, MAB10706 | WB | Mouse pAb |
| anti-TRIM59 | A E3 ligase | Thermo Fisher, PA5-106512 | IP | Rabbit pAb |
| anti-VPS39 | VPS39 subunit of HOPS complex | GeneTex, GTX32005 | IP | Rabbit pAb |
| anti-XBP1s | ER stress | Cell Signaling, 40435 | WB | Rabbit mAb |

**Note:** GAPDH, Glyceraldehyde 3-phosphate dehydrogenase; IFA, immunofluorescence assay; WB, western blot; IP, immunoprecipitation assay.
